# Loss of QKI in macrophage aggravates inflammatory bowel disease through amplified ROS signaling and microbiota disproportion

**DOI:** 10.1038/s41420-021-00444-w

**Published:** 2021-03-23

**Authors:** Wenwen Wang, Dongsheng Zhai, Yongquan Bai, Ke Xue, Lele Deng, Lirong Ma, Tianshu Du, Zicheng Ye, Di Qu, An Xiang, Guo Chen, Yi Zhao, Li Wang, Zifan Lu

**Affiliations:** 1 PLA Institute of State Key Laboratory of Cancer Biology, Department of Biopharmaceutics, Air Force Medical University, No. 17, Changle West Road, Xincheng District, Xi’an, Shaanxi Province China; 2 Department of Pharmacology, School of Pharmacy, Air Force Medical University, Xi’an, Shaanxi China; 3 Department of Dermatology, Xijing Hospital, Air Force Medical University, Xi’an, China; 4 PLA Institute of Orthopaedics, Xijing Hospital, Air Force Medical University, No. 17, Changle West Road, Xincheng District, Xi’an, Shaanxi Province China; 5 Breast Disease Diagnosis and Treatment Center of Affiliated Hospital of Qinghai University & Affiliated Cancer Hospital of Qinghai University, Xining, Qinghai Province China

**Keywords:** RNA modification, Inflammatory bowel disease

## Abstract

Inflammatory bowel disease (IBD) is a refractory chronic inflammatory illness of the gastrointestinal (GI) tract. Macrophage exerts an important role in IBD development. QKI, as an RNA binding protein, was related with inflammatory responses in bacterial infections by regulating the polarization of macrophages. Therefore, we suspected that QKI-regulated macrophages have the potential to play a certain role in IBD and the underlying mechanism. Our results demonstrated that the mice with macrophage-specific deletion of *QKI* induced with dextran sodium sulfate (DSS) are more susceptible to IBD development, exhibited a severe leaky gut barrier phenotype and higher intense oxidative stress, which are rescued by treating with butylated hydroxyanisole (BHA), an agonist of NRF2. Mechanically, we observed that *Keap1* mRNA in the nucleus was exported to the cytoplasm after LPS stimuli in parallel with QKI reductions, and the removal of QKI by shRNA facilitated *Keap1* mRNA nuclear exporting and expression in cytoplasm, consequently NRF2 activation in nucleus was weakened, and led to the impaired antioxidant abilities. In addition, mice models of fecal microbiota transplant (FMT) and the co-culturing of mice epithelia cells with feces derived from the DSS-treated QKI-deficit mice revealed consistently aggravated colitis along with a severe oxidative stress; 16S sequencing analysis substantiated the altered compositions of commensal bacteria too. Overall, the current study represents the first effort to explore the anti-oxidant role of QKI in the intestinal macrophage via post-transcriptional regulation of *Keap1* mRNA localization and the relevant NRF2 antioxidant signaling, and the disproportional changes in the microbiota were attributable to the mediation of pathogenic damage in the IBD development of QKI-deficit mice.

## Introduction

Inflammatory bowel disease (IBD) is a refractory chronic nonspecific inflammation disease of the colon and small intestine, including ulcerative colitis (UC) and Crohn’s disease (CD)^1^. Accumulating evidence supports that epithelial defects with the mucus layer destruction, disturbance of commensal or pathogenic bacteria, as well as immune imbalance together contribute to the development of IBD^2–5^. Conventional treatments including anti-inflammatory and immunosuppressive therapies fail to prevent the deterioration of IBD^6^. Therefore, better understanding the pathology and therapeutic scheme of IBD is urgently required.

In the gastrointestinal (GI) hollow organ, the intestinal epithelia act as a physical barrier that separates the intestinal microbiota from the underlying tissues to prevent bacterial infiltration and subsequent inflammation^7,8^. The structural and functional integrity of epithelial cells is extremely important for maintaining the tolerance of underneath immune system with symbiotic microorganisms. The disruption in the homeostasis of the immune system with microorganisms may induce epithelial cell destructions^9^.

Within the epithelial layer, macrophages-induced innate immune responses are the first line of defense against the invading pathogens^10–12^. It is well known that inefficient and overactivation of the GI macrophage subpopulation involved in the development of IBD are regulated by the process of initiation, amplification, and resolution of local inflammation^13,14^. During inflammation, oxidative stress is induced by the production of various free radicals, such as ROS (reactive oxygen species) and RNS (reactive nitrongen species), which are regarded not only as the etiological factors but also as the decisive ones in the progress of inflammation^15^.

Macrophages and neutrophils are considered as the main sources of ROS and RNS productions during intestinal inflammation. NAD(P)H oxidases (NOX1 and NOX2) are responsible for the generation of superoxide H_2_O_2_ in vivo. ROS generated by NOX2 strongly promotes macrophage activation, which is indispensable for the killing of microorganisms^16^. In contrast, the nuclear factor erythroid 2-related factor 2 (NRF2), as a key transcriptional regulator of the anti-oxidization process, usually mediates the transcriptional activations of various anti-oxidative protein expressions, such as those of GPX2, HO1, NAD(P)H, and NQO1^17^. At rest state, NRF2 is suppressed by Kelch-like erythroid cell-derived protein with CNC homology-associated protein 1 (KEAP1)-dependent ubiquitination-proteasomal degradation. Inflammation or other stress signals result in nuclear NRF2 activation by dislocating KEAP1 from the degradation complex due to the modifications of the oxidants and electrophiles^17,18^. Therefore, the precise regulation of NRF2 and KEAP1 is critical to maintain the balance between oxidative stress and intestinal homeostasis^19^.

RNA-binding protein quaking (QKI), a member of the Signal Transduction and Activator of RNA (STAR) family, is involved in post-transcriptional regulation of various mRNA expressions^20,21^. Previous studies have shown that QKI delays macrophage differentiation through negative feedback regulation of CCAAT/enhancer-binding protein (C/EBP) α^22^. Our previously study showed that QKI has the capability to modulate the polarization states of macrophages and regulate the severity of inflammation through the Ahr/NF-κB pathway in LPS (Lipopolysaccharide)-induced septic shock model^23^. However, the function and mechanism of QKI in the development of IBD have not been clarified.

In this study, we focused on the function of QKI in the activation of intestinal macrophages, particularly in IBD development, and the relevant underlying mechanisms. In the dextran sodium sulfate (DSS)-induced IBD mouse model, our finding showed that the GI barrier was heavily destroyed in QKI-deficient mice. In line with the higher expression of ROS and iNOS in QKI-deficit DSS models and the rescued phenotypes by the NRF2 agonist BHA, further molecular mechanisms can be related to KEAP1–NRF2 signaling mediated by QKI. Meanwhile, the composition of the microbiota significantly altered in the QKI-deficient mice.

## Results

### QKI knockout mice are more susceptible to DSS-induced intestinal inflammation

**Fig. 1:**
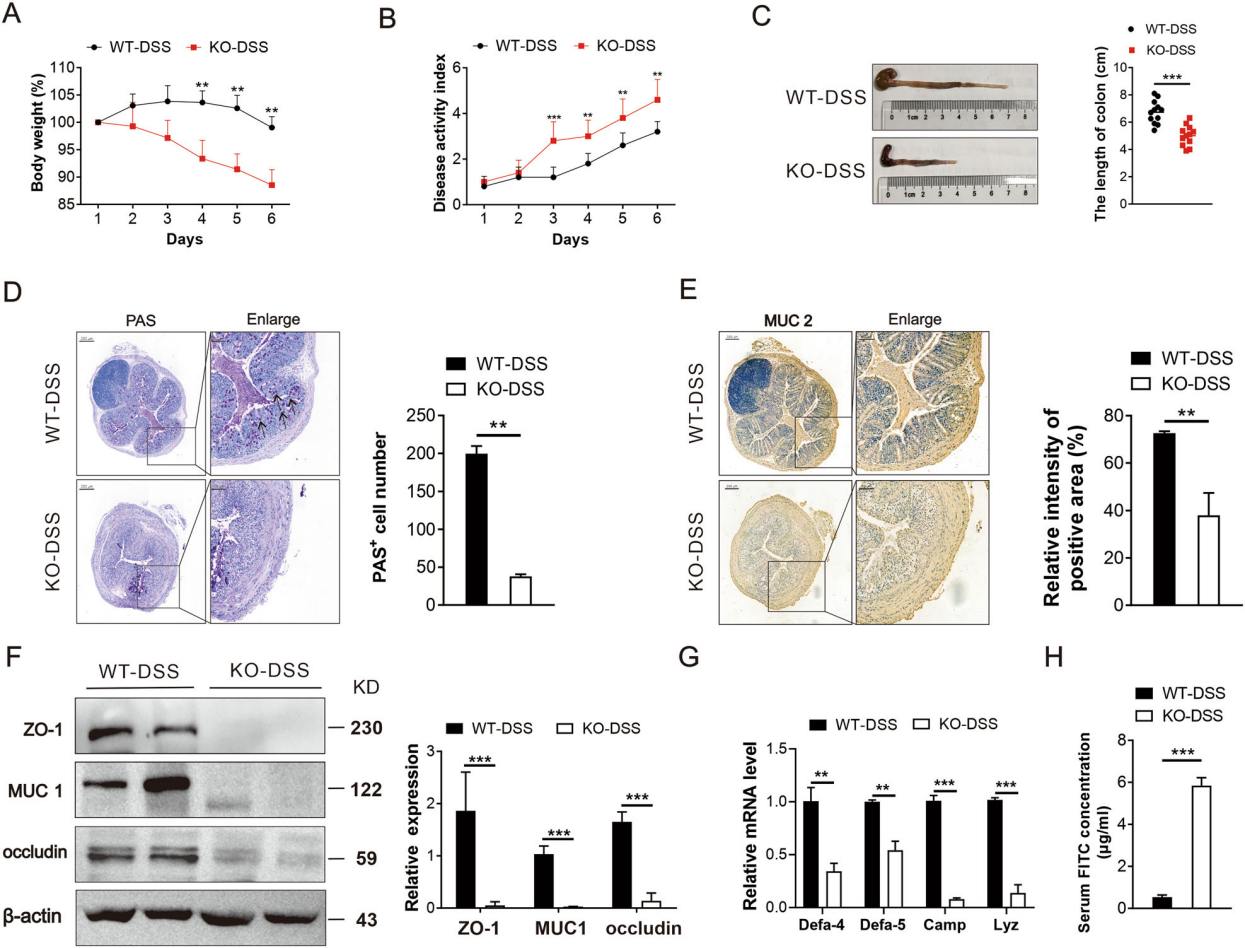
KO mice are susceptible to DSS-induced intestinal inflammation. QKI^fl/fl^ (WT) and LysM-Cre QKI^fl/fl^ (KO) mice were administered 3% DSS for 6 days to induce acute colitis. Mice body weight change (**A**) and disease activity index (DAI) (**B**) were assessed daily as described in “Methods” (*n* = 12/group). **C** Gross morphology of colons from WT-DSS and KO-DSS mice. The length of colon was measured on day 6 (*n* = 12/group). **D** Representative images of periodic acid-Schiff (PAS)-stained colons and statistical analysis the PAS-positive cells number in colon. Black arrows pointed to the PAS-positive cells. Scale bars: 200 μm (whole colon sections) and 50 μm (enlarged insets). **E** Expression of MUC2 in the colon was evaluated using immunohistochemistry. Representative images of MUC2 expression in mice colon were shown. The staining intensity of positive area of MUC2 expression was calculated by the percentage of positive area. Scale bar: 200 µm (whole colon sections) and 50 μm (enlarged insets). **F** Western blotting analysis of the protein expression of MUC1, ZO-1, and occludin in the colon of WT-DSS and KO-DSS groups. **G** mRNA level of antimicrobial peptides including *Defa-4*, *Defa-5*, *Camp*, and *Lyz* were detected by q-PCR. **H** Serum FITC concentration was detected after the mice were orally administered with FITC-labeled dextran (*n* = 5/group). All bars represent the mean of measurements from three independent experiments, and the error bars indicate ±SEM; *P < 0.05, **P < 0.01, ***P < 0.001, by unpaired, two-tailed Student’s *t* test (**A**–**H**).

In order to explore the potential role of QKI in the pathogenesis of IBD, we studied the QKI expression in the intestinal macrophages of active lesions and uninflamed mucosa from patients with UC and CD. Increased number of CD68^+^ QKI^+^ monocytes was observed in the inflammation lesions (Supplementary Fig. 1A, B). Moreover, the expression of QKI in CD11b^+^F4/80^+^ monocytes was significantly increased in the DSS-induced colitis mouse models, which was detected by flow cytometry (Supplementary Fig. 1C). These findings suggested that QKI may exert a functional role in the progression of IBD. QKI conditional knockout mice (LysM-Cre QKI^fl/fl^, KO) were generated and confirmed with macrophage-specific reductions of QKI as previously reported^23^. Firstly, we examined the weight and colons of KO mice under the H_2_O-fed, whereas no significant difference was detected between the KO mice and the control mice (QKI^fl/fl^, WT) (Supplementary Fig. 2A–D). Next, we evaluated the susceptibility of QKI-deficient mice to DSS-induced colitis (WT-DSS; KO-DSS). As shown in Fig. 1A, the body weights of KO-DSS mice were significantly lower than those in the WT-DSS mice 4 days after 3% DSS administration. The KO-DSS mice exhibited a significant increase in the disease activity index (DAI) score as compared with the WT-DSS mice 3 days after DSS administration (Fig. 1B). The colon length of the KO-DSS mice was markedly shorter than that of the WT-DSS mice (Fig. 1C). Goblet cells are an important constituent of the gut barrier responsible for the secretions of antimicrobial peptides and mucins. Periodic acid-Schiff (PAS) staining revealed that the number of goblet cells in KO-DSS mice was significantly reduced, which is a positive signal indicating polysaccharide production (Fig. 1D). Moreover, the diminished MUC2 levels were also observed after DSS treatment (Fig. 1E). As shown in Fig. 1F, the protein levels of tight-junctions (e.g., occludin and ZO-1) and MUC1 were markedly decreased in KO-DSS mice. The KO-DSS mice demonstrated a significant decrease in the mRNA expression of antimicrobial peptides as compared with WT-DSS mice, including *Defa-4*, *Defa-5*, *Camp*, and *Lyz* (Fig. 1G). Consistent with these molecular changes, the integrity of the gut was damaged, as evidenced by higher serum level of fluorescence intensity in FITC of KO-DSS mice after oral absorption of FITC-labeled dextran (Fig. 1H). These findings cumulatively indicate that the KO-DSS mice are more prone to intestinal injury in a DSS-induced IBD model.

### QKI deletion elevated the proportion of M1 macrophages in the colon

**Fig. 2:**
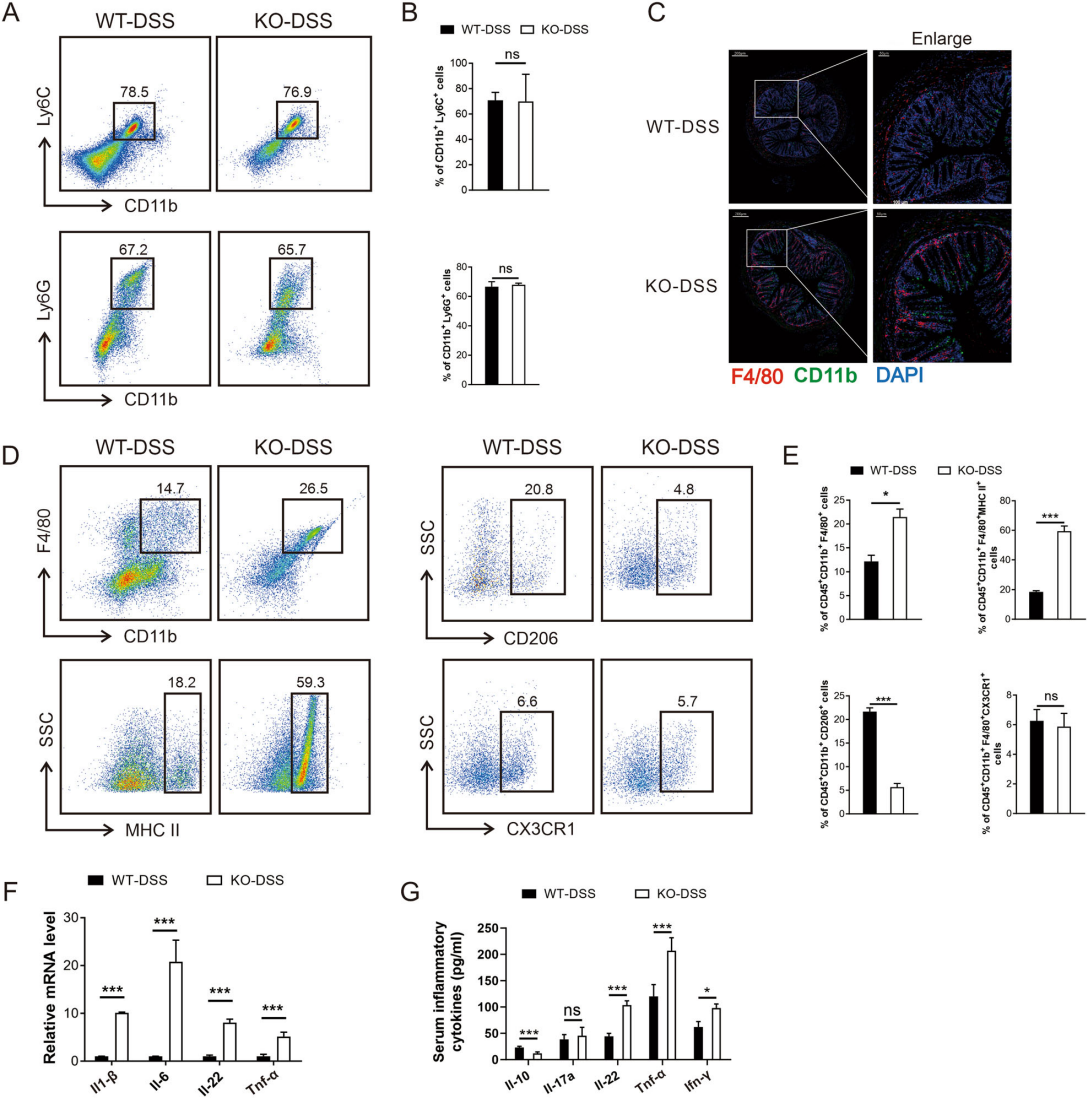
QKI deletion elevated the proportion of M1 macrophages in the colon. Lymphocytes were sorted from colonic lamina propria (CLP) of WT-DSS and KO-DSS mice and then gated from CD45^+^ group (*n* = 6/group). **A** The monocytes (CD11b^+^Ly6C^+^) and neutrophils (CD11b^+^Ly6G^+^) were identified by flow cytometry. **B** The percentage of labeled WT-DSS and KO-DSS monocytes and neutrophils were shown in a summary graph. **C** F4/80 (red), CD11b (green), and DAPI for nuclei (blue) in colon tissues from WT-DSS mice and KO-DSS mice. Scale bars: 200 μm (whole colon sections) and 50 μm (enlarged insets). **D** The percentages of macrophages (CD45^+^CD11b^+^F4/80^+^) were analyzed by flow cytometry. Then the M1-type macrophages (CD11b^+^F4/80^+^MHC II^+^), M2 macrophages (CD11b^+^F4/80^+^CD206^+^), and residential macrophages (CD11b^+^F4/80^+^CX3CR1^+^) were gated from CD45^+^CD11b^+^F4/80^+^ macrophages. **E** Qualification of macrophages, M1 macrophages, M2 macrophages, and residential macrophages. **F** q-PCR analysis the mRNA level of cytokines *Il-1β*, *Il-6*, *Il-22*, and *Tnf-α* in colon tissue. **G** The expression level of inflammatory cytokines including Il-10, Il-17a, Il-22, Tnf-α, and Ifn-γ in peripheral blood serum of mice were detected by ELISA. All bars represent the mean of measurements from three independent experiments, and the error bars indicate ±SEM; *P < 0.05, **P < 0.01, and ***P < 0.001, “ns” indicates not significant, by unpaired, two-tailed Student’s *t* test (**B**, **E**, **F**, **G**).

In adults, macrophages in the colon are mostly derived from peripheral monocytes and differentiate into different types of cells in the diverse state of colon^24^. No significant difference was observed in the percent of myeloid subsets between the two groups, including CD11b^+^Ly6C^+^ cells and CD11b^+^Ly6G^+^ cells (Fig. 2A, B). Immunofluorescence analysis showed that the recruited macrophages (CD11b^+^F4/80^+^) were significantly elevated in the colon of KO-DSS mice (Fig. 2C), indicating an altered immune environment in vivo. Furthermore, the percentages of total macrophages (CD45^+^CD11b^+^F4/80^+^) and M1-type macrophages (CD45^+^CD11b^+^MHCII^+^) were increased, whereas the percentage of M2-type macrophages (CD45^+^CD11b^+^CD206^+^) was decreased in QKI-deficient mice (Fig. 2D, E). CX3CR1 is a maker of residential macrophages in the mucosa^25^. Lack of QKI expression exerted no effect on the typical resident intestinal macrophage (CD45^+^CD11b^+^F4/80^+^CX3CR1^+^) population (Fig. 2D, E).

In terms of adaptive immunity, T cell subtypes in the inflamed tissues of colon in mice were examined after DSS treatment. As shown in Supplementary Fig. 2E, no significant difference was detected in the CD4^+^ and CD8^+^ T cells between the two groups. In comparison with WT-DSS mice, KO-DSS mice showed no significant change in the percent of Th1 cells (IL-2^+^CD4^+^), Th17 cells (IL-17^+^CD4^+^), and Treg cells (CD4^+^FOXP3^+^) (Supplementary Fig. 2F). A significant increase in the gene expression of *Il-1β, Il-6, Il-22*, and *Tnf-α* was detected in the colon of KO-DSS mice. The serum levels of cytokines, including Il-22, Tnf-α, and Ifn-γ, were also increased, while the level of anti-inflammatory cytokine Il-10 was reduced (Fig. 2F, G).

KO-DSS mice showed altered intestinal macrophage subsets with excessive activation of the pro-inflammatory responses, failing to influence the intestinal residential macrophage and adaptive immune T-cell populations during the acute period of colitis. These results suggest that QKI-deficiency macrophage is an important contributing factor leading to a more severe phenotype in KO-DSS mice.

### QKI deletion in macrophages caused excessive oxidative damage through the regulation of NRF2-related anti-oxidant pathways

**Fig. 3:**
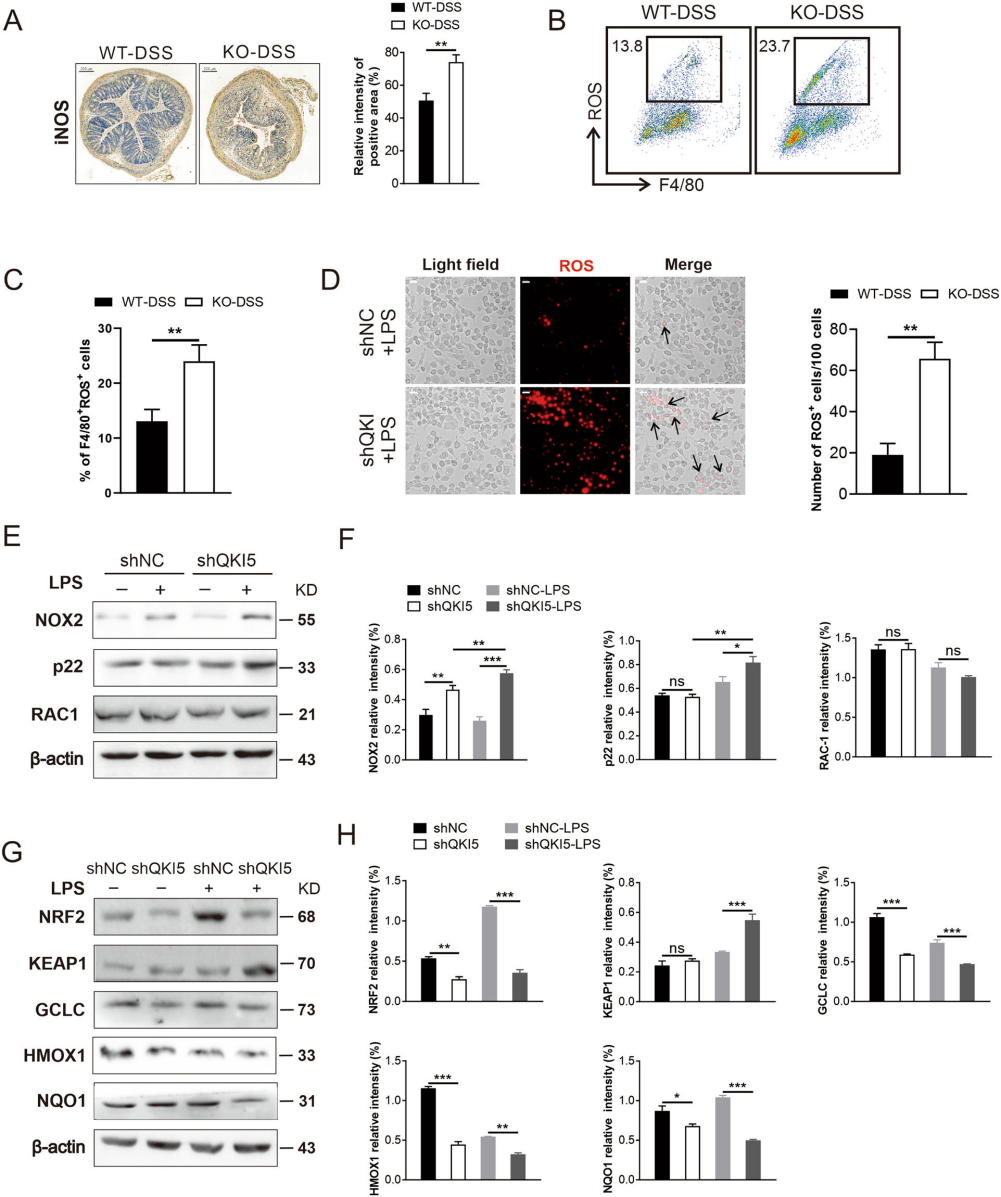
QKI deletion in macrophages caused excessive oxidative damage through the regulation of NRF2-related anti-oxidant pathways. **A** Representative images of IHC staining of iNOS in colon tissue of WT-DSS and KO-DSS mice. Scale bars: 200 μm. The staining intensity of the positive area (include mucosa and CLP) was quantified by ImageJ software (National Institutes of Health, Bethesda, MD) and illustrated with bar charts (*n* = 3/group). **B** The percentage of ROS^+^ macrophages collected from CLP of WT-DSS and KO-DSS groups were detected by flow cytometry and the cells were gated from CD45^+^CD11b^+^. **C** Statistical results of macrophages with high level of ROS. **D** The level of ROS of RAW 264.7 cells with QKI5 knockout (shQKI5, and shNC was a scramble control) which stimulated by LPS (100 ng/mL) for 24 h were detected by ROS probe (red). Scale bars: 20 μm. **E** NOX2, p22, and RAC1 proteins expression in shNC and shQKI5 cells treated with LPS for 24 h evaluated by western blotting. **F** Quantification of the band intensity of NOX2, p22, and RAC1. **G** Western blotting analysis of the expression of NRF2, KEAP1, GCLC, HMOX1, NQO1 in control and shQKI5 cells treated or not with LPS for 24 h. **H** Quantification of the band intensity of NRF2, KEAP1, GCLC, HMOX1, and NQO1. All bars represent the mean of measurements from three independent experiments, and the error bars indicate ±SEM; *P < 0.05, **P < 0.01, ***P < 0.001, “ns” indicates not significant, two-tailed Student’s *t* test (**A**, **C**, **D**) and one-way ANOVA with Tukey’s multiple comparisons (**F**, **H**).

Oxidative stress often occurs in the luminal environment when it interfaces with various organisms^26^. Macrophage is the vital source of luminal oxidants, including NO, iNOS, and ROS^27^. Remarkably high levels of iNOS in the colon of KO-DSS mice (Fig. 3A) and the UC and CD patients (Supplementary Fig. 3A, B) were observed based on immunohistochemical (IHC) staining. Although the ROS level of macrophages of colon tissue from KO-DSS mice was higher than that of WT-DSS mice (Fig. 3B, C), no significant difference was observed between the two groups when fed with H_2_O (Supplementary Fig. 3C, D). QKI5 silencing in RAW 264.7 cells was achieved and the efficacy of QKI5 dysregulation was confirmed by western blotting as previously described^23^. Results of ROS fluorescence staining revealed much higher ROS levels in shQKI5 cells with LPS treatment (Fig. 3D and Supplementary Fig. 3E, F). When the epithelial cell line MODE-K^28^ was co-cultured with the supernatant from shQKI5-LPS cells, the ROS level in MODE-K was increased. (Supplementary Fig. 3G, H).

As it is well known, the NADPH oxidase complex consists of NOX2, a transmembrane protein that assembles with various other proteins, including p22^phox^, p47^phox^, p67^phox^, p40^phox^, Rac1, and Rac2 (refs. ^17,29^). The assembled complex on the membrane of phago-lysosome is essential for ROS production^30^. Western blotting indicated a slight increase in the NOX2 and p22 expression in shQKI5 cells in comparison with shNC cells after LPS stimulation (Fig. 3E, F).

NRF2, a member of basic region leucine zipper (bZip) transcriptional factors^31^, confers resistance to oxidative stress^32^; and negatively regulated by KEAP1 (ref. ^19^) via ubiquitin-mediated degradation under normal conditions. In this study, after QKI silencing, the expression of NRF2 was significantly lower than that in shNC cells, especially under LPS stimulation. Similarly, the expression of other NRF2 downstream anti-oxidant target proteins, including HMOX1, NQO1, and GCLC, was significantly decreased in shQKI5 cells (Fig. 3G, H).

Overall, these results indicate that excessive oxidant production contributes to impaired intestinal barrier function in KO-DSS mice, which might be regulated by QKI in macrophage through the NRF2 anti-oxidant pathways.

### QKI directly affected *Keap1* mRNA localization and protein expression

**Fig. 4:**
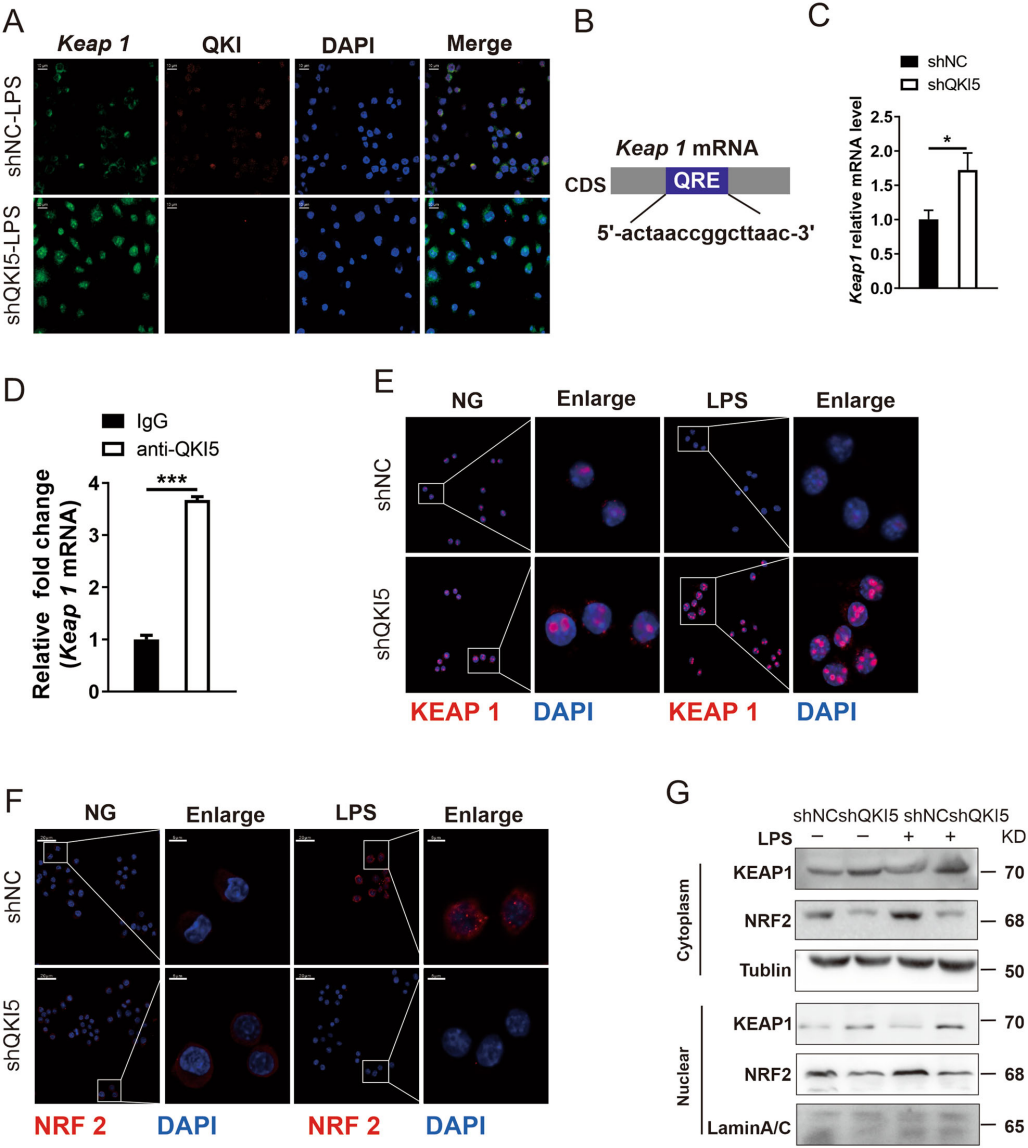
QKI directly affected Keap1 mRNA localization and protein expression. **A** RNA FISH was carried out to detect the interaction between QKI protein and *Keap1* mRNA. ShNC and shQKI5 cells were stimulated with LPS (100 ng/mL) for 24 h and then hybridized both with *Keap1* mRNA interaction probes (green) and QKI antibody (red). Nuclei are stained with DAPI (blue). Scale bar: 10 μm. **B** Bioinformatics analysis of the QKI binding site in the 3′UTR-to-CDS of *Keap1* mRNA. Regions corresponding to a potential QKI response element (QRE) sequence are shown in shaded boxes. **C** q-PCR detected the mRNA expression level of *Keap1* mRNA in shNC and shQKI5 cells. **D** RNA immunoprecipitation analysis using QKI antibody followed by real-time PCR to measure the mRNA levels of *Keap1* in QKI overexpressed RAW 264.7 cells (LV-QKI5, and LV-Cherry was a control) treated by LPS for 24 h, with β-actin used as a normalization control. **E**, **F** Confocal images of shNC and shQKI5 cells that were stimulated with LPS for 24 h and labeled with Abs against the appropriate protein. NRF2 and KEAP1 staining are shown in red and DAPI shown in blue. Scale bar, 20 µm and 5 μm (enlarge). **G** Western blotting analysis the expression of KEAP1 and NRF2 in lysates of cytoplasm and nuclear protein. All bars represent the mean of measurements from three independent experiments, and the error bars indicate ±SEM; *P < 0.05, **P < 0.01, ***P < 0.001, two-tailed Student’s *t* test (**C**, **D**).

Regarding the underlying molecular mechanisms in the above-mentioned NRF2 pathway, the nuclear co-localizations of QKI protein with mRNA of *Keap1* was detected by RNA fluorescence in situ hybridization (RNA-FISH). The results demonstrated that *Keap1* mRNA was co-localized with QKI protein. When QKI5 was silenced, *Keap1* mRNA started translocating from the nucleus into the cytoplasm in resting state (Supplementary Fig. 4A). After LPS stimulation, *Keap1* mRNA in nuclear was obviously exported to the cytoplasm in shQKI5 cells when compared with shNC cells (Fig. 4A). The bioinformatic analyses predicted that the coding region of *Keap1* mRNA contained QRE motif (Fig. 4B), and the total mRNA expression of *Keap1* was increased when QKI5 was silenced (Fig. 4C). The RNA immunoprecipitation (RIP) analysis demonstrated a specific interaction between the anti-QKI antibody and *Keap1* mRNA in comparison to IgG (Fig. 4D). Moreover, LPS treatment induced a significant increase in the NRF2 expression in shNC cells, which was greatly inhibited in shQKI5 cells (Fig. 4E). In contrast, the expression of KEAP1 protein was markedly increased in shQKI5 cells (Fig. 4F). The distributions of NRF2 and KEAP1 in nucleus and cytoplasm were further determined. As shown in Fig. 4G, the expression of NRF2 was elevated in nucleus and cytoplasm under LPS stimulation in shNC cells, which was barely detectable after silencing QKI5. However, the expression of KEAP1 was increased after LPS stimulation in both the nucleus and cytoplasm of shQKI5 cells.

Taken together, these results show that QKI5 can control *Keap1* expression via trapping its mRNA in the nucleus, and the removal of QKI5 can facilitate the export of *Keap1* mRNA to the cytoplasm and the subsequent protein translation.

### BHA supplementation rescues the damage of the colon in KO-DSS mice

**Fig. 5:**
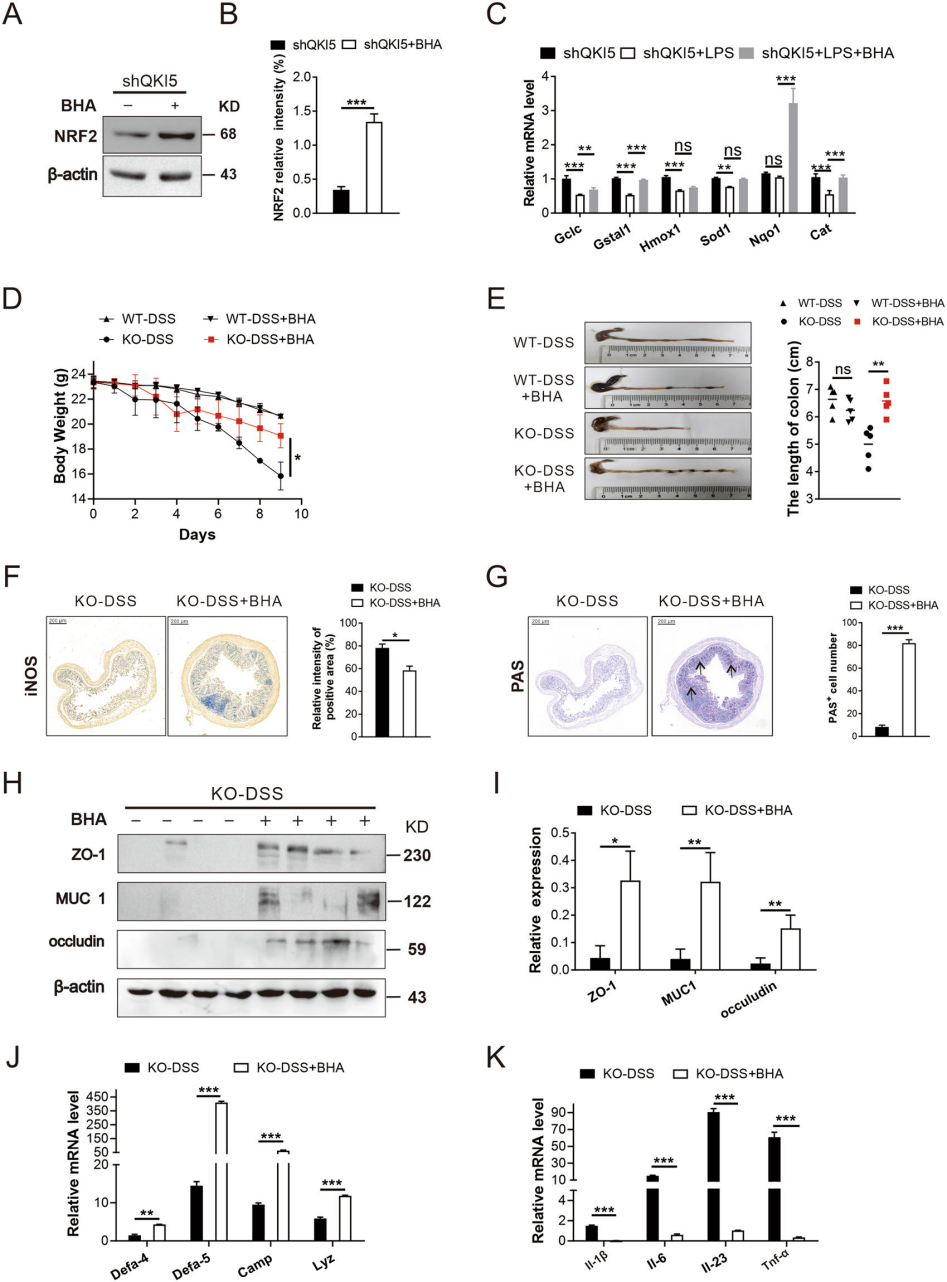
BHA supplementation rescues the damage of the colon in KO-DSS mice. **A** NRF2 protein expression in shQKI5 cells, after treating with BHA (100 μM) for 4 h, was detected by western blotting. **B** Qualification of the band intensity of NRF2. **C** q-PCR detected the mRNA expression level of *Gclc*, *Gsta1*, *Hmox1*, *Sod1*, *Nqo1*, and *Cat* after BHA treatment. **D** Mice of WT and KO were randomly divided into two groups (*n* = 5/group) and one group was administered with 3% DSS in drinking water for 7 days after receiving BHA (200 mg/kg) for 3 days by oral gavage while the other group was just treated with 3% DSS for 7 days. Body weight changes of each group were recorded daily. **E** Morphology of colons from WT and KO mice with or without BHA treatment. Colon lengths were measured on day 9. **F** Representative iNOS immunohistochemical staining of colon specimens obtained from KO mice with BHA treatment. Scale bars: 200 μm. **G** PAS-stained images of colon sections. Black arrows pointed to the PAS-positive cells. Scale bars: 200 μm. **H** Occludin, MUC1, and ZO-1 proteins from control mice and mice after BHA treatment were analyzed by western blotting. **I** Qualification of the band intensity of occludin, MUC1, and ZO-1 from three independent experiments. **J** q-PCR analyzed the mRNA expression level of *Defa-4*, *Defa-5*, *Camp*, and *Lyz* in colon tissue after BHA treatment. **K** q-PCR analyzed the mRNA expression level of *Il-1β*, *Il-6*, *Il-23*, and *Tnf-α*. All bars represent the mean of measurements from three independent experiments, and the error bars indicate ±SEM; *P < 0.05, **P < 0.01, ***P < 0.001. Two-tailed Student’s *t* test (**B**, **C**, **F**, **G**, **I**–**K**) and one-way ANOVA (**D**, **E**).

For further verification of the function of NRF2-related pathway in the aforementioned regulation, BHA (an agonist of NRF2) was used. The expression of NRF2 was significantly elevated in shQKI5 cells after BHA treatment (Fig. 5A, B and Supplementary Fig. 4B). Simultaneously, several transcripts of downstream genes were increased, including *Gclc* and *Gstal* (Fig. 5C). Moreover, BHA remarkably suppressed the ROS overproduction in shQKI5-LPS cells (Supplementary Fig. 4C). Next, WT-DSS and KO-DSS mice were treated with BHA. Administration of BHA could restore the body weight in KO-DSS mice but there was no significant change in WT-DSS mice (Fig. 5D). The length of the colon in KO-DSS mice was longer after the BHA administration (Fig. 5E). The expression of iNOS was decreased in KO-DSS mice as compared with WT-DSS mice after BHA treatment (Fig. 5F). Moreover, the colon histology revealed that the number of PAS-positive cells was significantly increased in KO-DSS mice (Fig. 5G). The protein levels of MUC1 and tight-junction (ZO-1, occludin) were higher in KO-DSS mice with BHA treatment (Fig. 5H, I). The mRNA levels of antibacterial peptides, including *Defa-5*, *Camp*, and *Lyz-1* were increased in KO-DSS mice (Fig. 5J). Furthermore, BHA significantly inhibited the mRNA expression of *Il-1β*, *Il-6*, *Il-23*, and *Tnf-α* in KO-DSS mice (Fig. 5K). Collectively, these data indicate that BHA exerts beneficial effects on severe colitis phenotype of KO-DSS mice by promoting the expression of NRF2 and improving the antioxidant stress ability of macrophage, which further verifies the role of NRF2 pathway.

### The gut microbiota derived from the KO-DSS mice displayed more severe colon injuries in the fecal microbiota transplant C57BL/6J mice

**Fig. 6:**
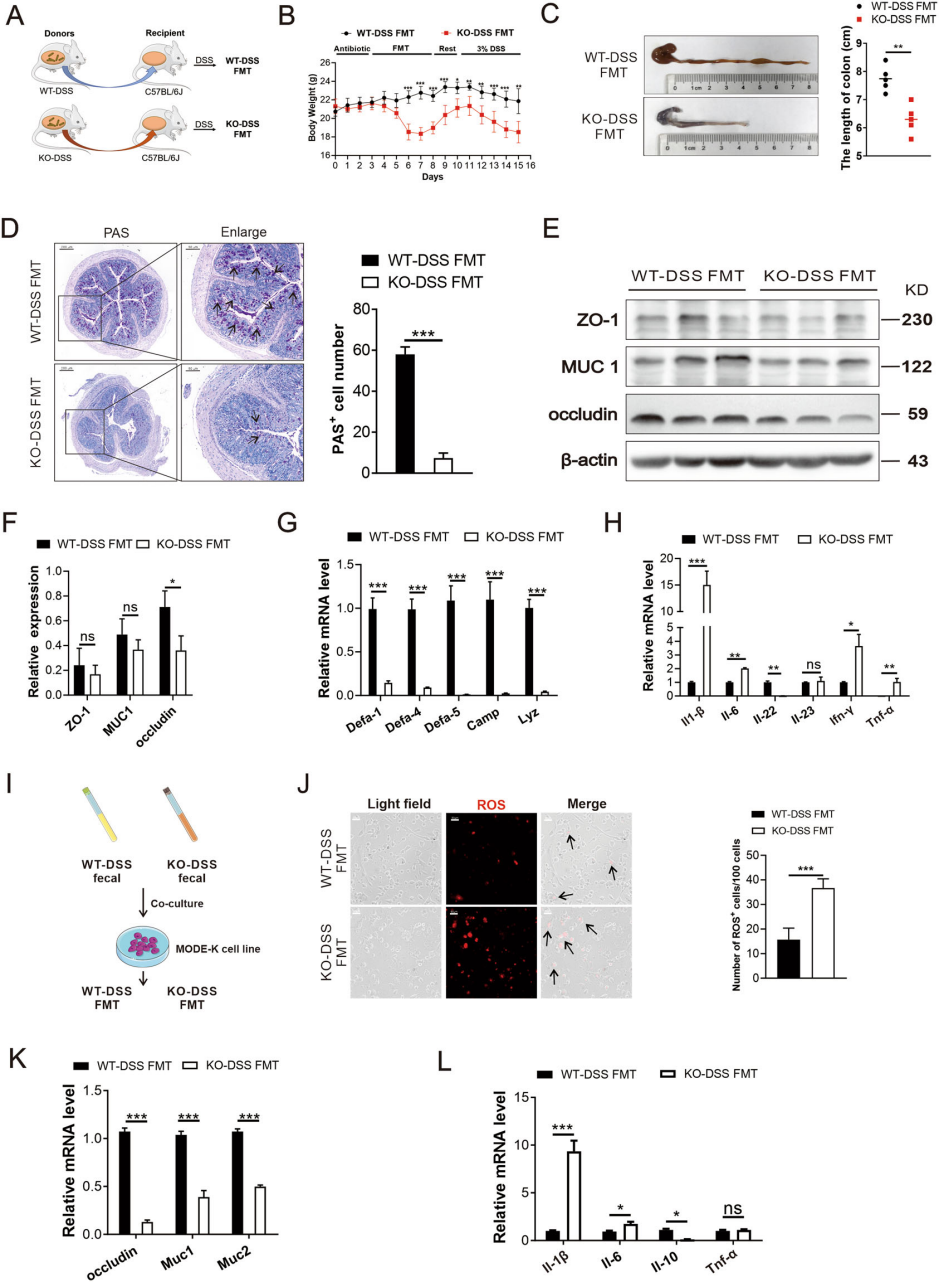
The gut microbiota derived from the KO-DSS mice displayed more severe colon injuries in the FMT C57BL/6J mice. **A** Mice were administered with a spectrum antibiotic mixture of penicillin (200 U/mL) and streptomycin (200 μg/mL) by oral gavage for 3 days and then transplanted with microbiota which were harvested from WT-DSS (WT-DSS FMT) and KO-DSS (KO-DSS FMT) mice for 5 days and rested for 3 days. Finally, 3% DSS was given to these mice and sacrificed in 7 days (*n* = 5/group). **B** Body weight was recorded during FMT assay. **C** The length of the colon from WT-DSS FMT and KO-DSS FMT mice was recorded after 7 days of 3% DSS treatment. **D** Representative images of PAS staining of the colon tissue and statistical analysis of the PAS-positive cells number. Black arrows pointed to the PAS-positive cells. Scale bars: 200 μm (whole colon sections) and 50 μm (enlarged insets). **E** The expression of ZO1, MUC1, and occludin in the colon of WT-DSS FMT and KO-DSS FMT mice were detected by western blotting. **F** Qualification of the band intensity of occludin, MUC1, and ZO-1. **G** q-PCR analyzed the mRNA expression level of antimicrobial peptides such as *Defa-1*, *Defa-4*, *Defa-5*, *Camp*, and *Lyz* in colon tissue. **H** q-PCR analyzed the mRNA expression level of *Il-1β*, *Il-6*, *Il-22*, *Il-23*, *Ifn-γ*, and *Tnf-α* in colon tissue. **I** Fecal microbiota from WT-DSS and KO-DSS mice co-cultured with intestinal epithelial cell line MODE-K. **J** ROS levels of MODE-K cells treated with microbiota were detected by fluorescent probe (red). The black arrows were directed toward the ROS-positive cells. Scale bars: 50 μm. **K** q-PCR analyzed the mRNA expression of *occludin*, *Muc1*, *Muc2* after MODE-K cells were infected with fecal microbiota. **L** q-PCR analyzed the mRNA expression of *Il-1β*, *Il-6*, *Il-10*, and *Tnf-α* in MODE-K infected with fecal microbiota. All bars represent the mean of measurements from three independent experiments, and the error bars indicate ±SEM; *P < 0.05, **P < 0.01, ***P < 0.001, “ns” indicates not significant. Two-tailed Student’s *t* test (**B**–**H**, **J**–**L**).

It has been shown that the inflammatory intestinal environment has the capability to influence the gut microbiota^33^. Similarly, we observed that a decrease in the expression of antimicrobial peptide-related mRNA (e.g., *Defa-1*, *Defa-4*, *Defa-5*, *Camp*, *Lyz*) has an effect on the microbiota in the colon of KO-DSS mice as compared with WT-DSS mice. Fecal microbiota transplant (FMT) experiment was designed to explore the potential effect of microbiota in WT-DSS and KO-DSS mice. C57BL/6J mice were given the fecal content of WT-DSS and KO-DSS mice by oral gavage, and subsequently fed with drinking water containing 3% DSS (Fig. 6A). Notably, the mice body weight in the KO-DSS FMT group was dramatically decreased in comparison to that in the WT-DSS FMT group, even without DSS treatment (Fig. 6B), and the length of the colon was shorter after the DSS treatment of KO-DSS FMT mice (Fig. 6C). Furthermore, PAS staining demonstrated more than fourfold decrease in cells positive for membrane-localized Villin of KO-DSS FMT group mice, suggesting a concomitant decrease in mucin-producing cells (Fig. 6D). The expression of MUC1 and occludin was also decreased in KO-DSS FMT group when compared with the WT-DSS FMT group (Fig. 6E, F). At the same time, the level of iNOS in KO-DSS FMT group was also higher than that of WT-DSS FMT group (Supplementary Fig. 4D). Compared with the WT-DSS FMT group, the mRNA expression of anti-bacterial peptides in the recipient of KO-DSS microbiota was decreased (Fig. 6G). Moreover, KO-DSS FMT group displayed higher levels of typical pro-inflammatory cytokines, including *Il-1β*, *Il-6*, and *Tnf-α* (Fig. 6H). To further validate the different pathogenic functions of feces between the two groups, we observed the effects of bacteria mixture on the small intestine epithelial cells (MODE-K cell line) (Fig. 6I). Interestingly, the intestinal microbiota from KO-DSS mice feces resulted in increased ROS production in MODE-K cells (Fig. 6J). Moreover, KO-DSS FMT group exhibited significantly lower expression of *occludin*, *Muc1*, and *Muc2* and significantly higher expression of pro-inflammatory *Il-1β* and *Il-6* as compared with WT-DSS FMT group. On the contrary, the expression of anti-inflammatory cytokine *Il-10* was decreased in the KO-DSS FMT group (Fig. 6K, L). Finally, the increased level of ROS was inhibited in MODE-K cells, which was obtained by co-culturing with microbiota derived from KO-DSS+BHA mice (Supplementary Fig. 4E, F).

Collectively, the above-mentioned data support that the microbiota contributed to the severe gut damage in KO-DSS mice. Moreover, the overproduction of pro-inflammatory cytokines and ROS between the microorganisms and intestinal epithelia cells may account for the defective integrity of the gut barrier.

### Altered compositional patterns of microbiota in KO-DSS mice

**Fig. 7:**
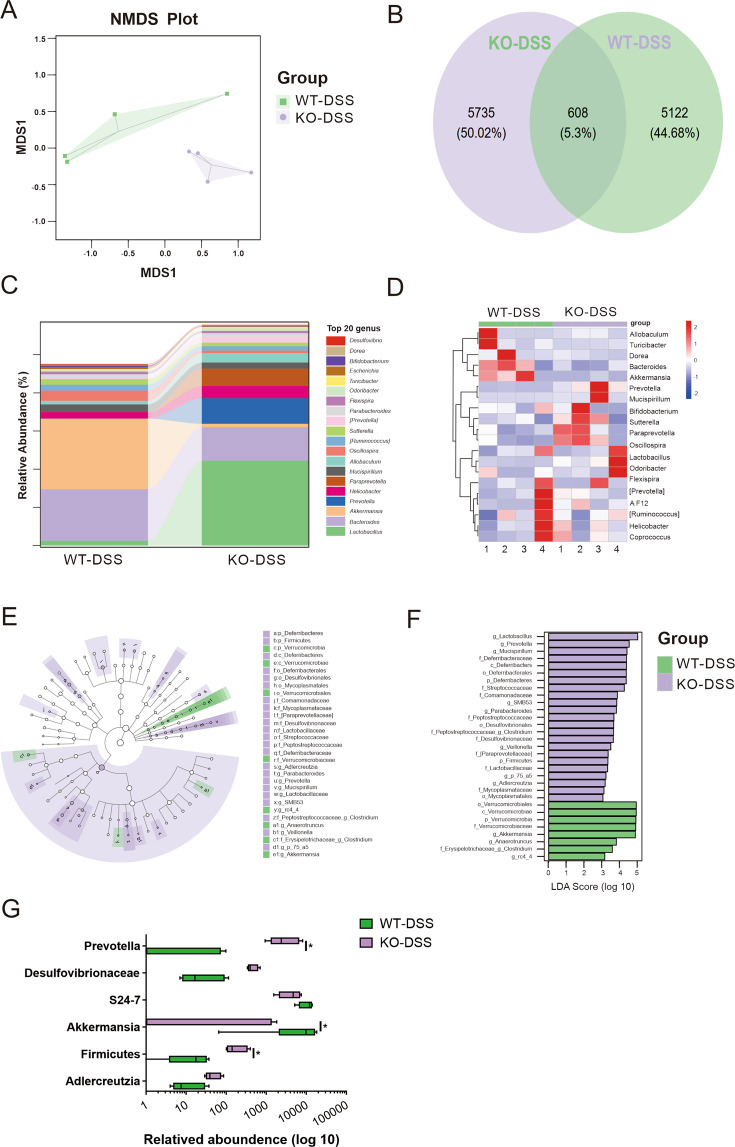
Altered compositional patterns of microbiota in KO-DSS mice. **A** Nonmetric multidimensional scaling analysis on OTUs (*n* = 4/group). Each sample represents a single sample (from one mouse) and is colored according to group. **B** The Venn diagram shows that OTUs were overlapped in each group. **C** Histogram of genus horizontal species composition between the two groups. **D** The heat map of genus level species composition shows abundance of the top 20 bacterial taxa. The red block indicates that the genus has a higher abundance in the sample than other samples, while the blue block indicates that the genus has a lower abundance in the sample than other samples. **E** Bar chart of LDA effect values of identified species. **F** Display diagram of inter-group difference taxon based on classification rank tree. **G** Relative abundance of each group. Statistical analyses were performed with **A** the Permanova test, or **G** the Mann–Whitney *U* test. NMDS, nonmetric multidimensional scaling.

To clarify whether the diversity and composition of gut commensal bacteria from KO-DSS mice were affected, which may aggravate IBD^34^, 16S ribosomal DNA sequencing was conducted to profile intestinal microbial diversity. Rare new phylotypes and most of the diversity in the Rarefaction and Shannon index analysis were covered by the sequencing depth (Supplementary Fig. 5A). Major differences in the microbial composition between WT-DSS and KO-DSS mice were observed after nonmetric multi-dimensional scaling analysis (Fig. 7A). Although no significant difference was noted in the diversity of gut microbiota between WT-DSS and KO-DSS group (Supplementary Fig. 5B), a statistically significant difference was observed in the operational taxonomic unit (OTU) profiling and unweighted UniFrac distance-based principal coordinate analysis shown in the composition of KO-DSS mice microbiota (Fig. 7B and Supplementary Fig. 5C, D). Detailed analysis of the top 20 genera of the gut microbiota indicated that KO-DSS mice had increased abundance of *Lactobacillus, Prevotella*, and *Turicibacter* species, but markedly reduced populations of *Akkermansia* and *Bacteroides* species when compared to WT-DSS mice, which were further confirmed in the heatmap (Fig. 7C, D). Moreover, marked differences were noted during a supervised comparison of the microbiota between WT-DSS and KO-DSS groups by using cladogram analysis (Fig. 7E) and the linear discriminant analysis effect size (LEfSe) tool (Fig. 7F). The statistical analysis of the difference in the taxa at the genus level revealed a significantly higher proportion of the genus *Prevotella*, *Desulfovibrionaceae*, *Firmicutes*, and *Adlercreutzia* and a lower proportion of *Akkermansia* and *S24-7* in KO-DSS group (Fig. 7G). All the results of sequencing analysis indicate that myeloid *QKI*-deficiency alters the proportion of microbiota in mice colitis, which shall contribute to the development of severe colitis phenotypes.

## Discussion

IBD is a chronic, relapsing illness that presents with an uncontrolled immune response against the GI tract^35^. Anti-inflammation therapies have been effectively applied on the clinical scale^36^. However, there is a need to clarify the underlying mechanisms of this disease in order to design the recurrence-prevention measures. Firstly, the current study demonstrated that LysM-Cre QKI^fl/fl^ mice exhibited a more severe colitis phenotypes after being administered with 3% DSS, featuring with the destroyed gut barrier and colonic epithelial cells, as well as the relevant changes in the molecular markers, whereas there are no significant differences in the normal conditions between the WT and KO mice in the adult stages. Secondly, we found that the population of total macrophage in the colon section was increased, especially M1 type. QKI deficiency in macrophage displayed a higher ROS level that may be related with the dampened anti-oxidant ability via regulating NRF2-KEAP1 pathway. Treatment with BHA, an agonist of NRF2, mitigates the severe IBD phenotype in QKI-deficit mice. Thirdly, the impairment of intestinal mucus layer, the higher levels of pro-inflammatory factors, as well as the reductions of anti-microbial peptides all resulted in disturbed intestinal microbiota. The complex interactions between disproportional microbiota and gut environments in FMT mice strongly supported that the disturbed microbiota exerted a crucial pathogenic role in IBD development.

DSS-induced IBD mice model is consistent with the pathological changes that occur in IBD patients, including exaggerated pro-inflammatory innate and adaptive immune responses and mucosal epithelial injury. By using the myeloid cell-specific QKI KO mouse treated with DSS, histological and functional characterizations disclosed that more severe leaky GI barrier occurred in QKI-deficit mice, indicated as a higher serum level of FITC-dextran from oral absorption. Tight-junction-related molecules were reduced, and number of PAS and MUC2 staining-positive goblet cells were both decreased, pointed a weakened functional condition of intestinal epithelial cells. In addition, the secretion levels of MUC1 and anti-bacterial peptides were remarkably decreased, demonstrating an impaired mucus layer, which is the mainly efficient system for protecting the epithelium from bacteria. The above-mentioned indicators support that the gut macrophage cell dysfunction is decisive in IBD development.

To explore the relevant immunological response mechanisms involved, we detected both the innate and adaptive immune cells in the colon. Interestingly, the total numbers of macrophages (CD45^+^CD11b^+^F480^+^), especially the ratio of pro-inflammatory subtype (CD45^+^CD11b^+^F480^+^MHCII^+^), were significantly increased, while the anti-inflammatory subtype (CD45^+^CD11b^+^CD206^+^) was reduced. Notably, the CD45^+^CD11b^+^F4/80^+^CX3CR1^+^ subset, defined as intestinal residential macrophages, did not change much. These data cumulatively suggest that the infiltration of the macrophages, which mobilized from the bone marrow, was increased in the colon tissue, and the group cells of polarization to M1 type was increased as well, accordingly with the result we detected in endotoxemia mouse model^37^. Therefore, QKI expression in macrophage exerts anti-inflammatory function to maintain the homeostasis of the GI tissues.

Notably, the severe IBD symptoms in terms of body weight, iNOS level, and leaky mucosal barrier are totally rescued by treatment with the anti-oxidant agent BHA, an NRF2 agonist^38^. Therefore, an intriguing question that remains to be answered is how QKI loss in macrophages amplifies the ROS levels. By using the in vitro cell models, we defined that QKI protein directly binds to *Keap1* mRNA and may trap *Keap1* mRNA in nucleus. Under LPS stimuli, QKI loss facilitated *Keap1* mRNA exporting from the nucleus to the cytoplasm, as detected by *Keap1*-FISH. Both immunofluorescence assay and western blotting results further demonstrated that NRF2 nuclear activation under LPS treatment was weakened by shRNA QKI5, while KEAP1 protein was more enriched in both cytoplasm and nucleus. Presumably, the higher KEAP1 in cytoplasmic or nuclear regions helps in degrading NRF2 via the ubiquitin system to prevent its activation. Thus, as far as we know, this is the first report showing that *Keap1* mRNA was regulated in the nucleus by QKI5. QKI5 reduction facilitates more *Keap1* mRNA exporting and protein expression in cytoplasm to suppress NRF2 activation. Following the abovementioned oxidative stress-induced amplified ROS signals, a severe pro-inflammatory state was released. As expected, treatment with the NRF2 agonist BHA recovered the nuclear NRF2 level and attenuate the deleterious effects of IBD phenotype in KO-DSS mice. These data collectively consolidate that enhancing the antioxidant ability is beneficial for IBD treatment.

Regarding *Keap1* mRNA regulation by QKI, besides the cytoplasmic KEAP1 level, the protein expression of nuclear KEAP1 is also increased after QKI loss. There is, however, no evidence that supports the function of nuclear KEAP1 protein, and we did not obtain direct evidences of alterations in *Keap1* mRNA translational efficiency either, which should be evaluated in the future research.

QKI-deficient mice with DSS induced the disproportional changes of microbiota which mediated severe DSS colitis in FMT mice. More recent evidences support that homeostasis of the microbiota is dynamically and strictly regulated under normal condition and that the disproportion of commensal bacteria is closely related with the development of various diseases, such as IBD, diabetes, cardiovascular illness, and cancers^39–41^. By administering FMT in mice, we observed that KO-DSS mice-derived FMT showed more severe body weight loss and IBD symptoms without or with the DSS treatment. Moreover, the in vitro cell co-culturing assays with microbiota and mice intestinal epithelial cell line MODE-K further demonstrated that there were higher ROS levels and more pro-inflammatory cytokines, including Il-1β, Il-6, and Tnf-α, and severe epithelial damages in the group from KO-DSS mice feces relative to the WT-DSS group. These evidences highly support that the disturbed microbiota contribute significantly to the development of severe leaky gut symptoms. The 16S sequencing analysis of microorganisms further disclosed the dramatic proportional changes in the microbiota genus. The latter genus is known to lead the result of anti-inflammatory effects in the host, as reported previously^42^. All in all, our results highly suggest that the disproportional changes of the microbiota directly affect the severity of IBD phenotype by enhancing the ROS stress in the epithelial layers. Although administering healthy FMT is fast gaining popularity as a novel strategy for disease control^39,43^, the safety and efficiency of this approach remain to be explored.

Overall, our study supported that the amplified ROS levels are the key signals mediating the pro-inflammatory phenotypes of QKI loss in macrophages, and more importantly, the disturbed microorganisms are also critical for mediating the gut pathology. The triad communications among innate immune cells, microbiota, and mucosal epithelia are interconnected with each other via exaggerated ROS signals during IBD development. Therefore, QKI-related antioxidant agents or healthy microbiota transplant may serve as potential effective intervening methods for the IBD disease control.

## Materials and methods

### Human tissue specimen

All the specimens were obtained from the pathology department of Xi’jing Hospital, the first affiliated hospital of the Air Force Military Medical University. The diagnosis of CD and UC was confirmed by established criteria of clinical, radiological, and endoscopic analyses, and from histology reports. Informed consent was obtained and procedures were performed according to approval by the local ethics committee of the Air Force Military Medical University.

### Animals and treatment

All animal experiments and procedures were approved by the Laboratory Animal Center of Air Force Military Medical University and conducted in compliance with the ethical standards. Macrophage-specific QKI-deletion mice (LysM-Cre QKI^fl/fl^, KO) were generated via an intercross between the QKI-floxed mice (QKI^fl/fl^, WT) and LysM-Cre mice. The KO and WT mice were administered with 3% DSS (ICN Biomedicals, Santa Ana, CA) for 7 days. The body weight, stool consistency, and stool bleeding parameters of the mice were recorded after 7 days and subjected to the calculation of the DAI value. Both the groups of mice (*n* = 6/group) received BHA (200 mg/kg, BW) via oral gavage daily for 3 days before the DSS treatment. BHA was dissolved in corn oil and the control group gavage with corn oil. After all the animals were sacrificed at the end of the experiment, the sections between the cecum and the proximal rectum were measured to obtain the colon length.

### Cell culture

The construction of shQKI5 or QKI5 overexpression in RAW 264.7 cells was established as reported previously^23^. Briefly, the cells were cultured in RPMI-1640 medium (Hyclone) with 10% FBS and grown at 37 °C in an incubator containing 5% CO_2_ atmosphere. BHA was dissolved in DMSO and then diluted to 500 mM using serum-free medium and co-cultured with cells for 12 h with the addition of LPS (100 ng/mL). MODE-K cells were cultured in DMEM medium (Hyclone) containing 10% FBS. When the microbiota-infected cells were collected, the cells were co-cultured with the bacterial solution resuspended in PBS for 1 h and then removed, and replaced with medium diluted in PBS for another 3 h.

### Isolation of lymphocytes from the colon

The colon lamina propria mononuclear cells (LPMCs) were isolated with enzymes. First, LPMC suspension was prepared. Briefly, large pieces of fat were removed without tearing the intestinal tissues. Then, the intestines were gently flushed with 15–20 mL of cold PBS to remove all feces. The colons were then chopped into 1-mm pieces, to which HBSS supplemented with 1-mm DTT (Sigma, USA) and 35-mmol EDTA were added, and the mixture was incubated on a shaker incubator for 20 min at 37 °C. Next, the liquid culture was passed through an 80-µM cell strainer and then the minced colon tissues were added to the supplemented HBSS buffer repeatedly. The residual colon tissues were incubated for 1 h at 37 °C in RPMI 1640 containing 100 U/mL Collagenase VIII (Sigma) and 150 µg/mL of DNAse1 (Sigma). The LPMC suspension was next passed through a 40-µM cell strainer (Corning, USA) and centrifuged at 550×*g* for 5 min. Percoll solution (Sigma) was used to isolate the lymphocytes, and the cells were stained with trypan blue to examine the cell viability and number.

### Flow cytometry and intracellular cytokine staining

In order to determine cytokine secretion, the prepared cell suspension was incubated with Cell Stimulation Cocktail (BD eBioscience, NSW, Australia) for 16 h. The cells were then collected, fixed, and permeabilized with IC Fixation Buffer (BD eBiosciences) and Permeabilization Buffer (BD eBiosciences). The resultant cell suspensions were stained with the surface fluorescent antigens by first blocking the cells with 0.5 × 10^6^ F_c_ (BD eBiosciences) with the following anti-mouse monoclonal antibodies: anti-mouse CD45 FITC, anti-mouse CD11b PE, anti-mouse F4/80 PE-Cy5, anti-mouse CD206 PE-594, anti-mouse CX3CR1 PE-Cy7, anti-mouse major histocompatibility complex II (MHC II) PE-Cy7, anti-mouse CD4 FITC, anti-mouse Ly6C PE-Cy7, and anti-mouse Ly6G FITC (all sourced from BD eBiosciences). ROS was detected by Reactive Oxygen Species (ROS) Detection Reagents (CM-H2DCFDA, Invitrogen). The Fixable Viability Dye eFluor^TM^ 780 (eBioscience) was used to exclude the dead cells. For intercellular staining, fixation, permeabilization, and staining were performed with the following fluorescent-labeled intracellular molecules antibodies: anti-mouse IL2 PE-594 (eBioscience), anti-mouse IL17 PE-Cy7 (ebio17B7; eBioscience), and anti-mouse Foxp3 PE (FJK-16s, eBioscience). Gallios (Beckman Coulter) was used in the transmigration assays and the data were analyzed by the FlowJo 10.4 or Kaluza software 2.1 (Beckman Coulter) for the transmigration assays.

### IHC staining

IHC staining was performed as per the manufacturer’s instruction. After paraffin-embedding, deparaffinizing, rehydrating, and restoring antigen, the sections were treated with 3% H_2_O_2_ and blocked with goat serum for 1 h at 37 °C. The sections were then incubated with MUC2 and iNOS antibodies overnight at 4 °C, followed by treatment with HRP-Polymer anti-Rabbit. The number of positive cells and the percent of positive area were calculated by the ImageJ 1.52v software (National Institutes of Health software, USA).

### Enzyme-linked immunosorbent assay

The concentrations of Tnf-α, Ifn-γ, Il-17a, and Il-22 in the serum and cell culture supernatant were determined by enzyme-linked immunosorbent assay (ELISA) (Biolegend, Dakewe, China) as per the manufacturer’s instructions. Antibody, standards, and samples were added to 96-well plates and co-incubated for 90 min. The antibody was added to the cells and incubated for 1 h, followed by the addition of Avidin-HRP for 30 min, and, finally, TMB solution for 10–15 min, all at 37 °C. Before the reaction was stopped, the co-incubation liquid was detected at 450 nM. The limitation of sensitivity was 15 pg/mL for TNF-α and IL-17a, and 3 pg/mL for IFN-γ and IL-22.

### Fecal microbiome transplantation

To remove the bacteria from the gut of SPF mice, penicillin (Sigma-Aldrich, 200 U/mL) and streptomycin (Sigma-Aldrich, 200 μg/mL), which are typical spectrum antibiotics, were adminitered via oral gavage to mice for 3 days. Feces were collected from WT-DSS and KO-DSS mice and stored at −80 °C until used. After being resuspended with 1.5 mL of PBS, filtered through a 100-μm nylon mesh strainer (Corning), the filtered contents were resuspended with PBS in total volume of 2 mL, of which 150 μL per mouse was administered to GF mice in the vinyl isolator via oral gavage lasting for 5 days, and then rest for 3 days before DSS treatment. When treated with cells, the microbiota was diluted and co-cultured with the cells for 4 h before examination.

### Western blotting

The total protein in the colon tissues was extracted with the RIPA buffer (Thermo-Fisher Scientific, NSW, Australia). The protein concentration was determined by the BCA assay (Thermo, UK) and equal amounts of proteins were run on 10% SDS polyacrylamide gels, followed by transferring them onto an NC membrane (Millipore, USA). After blocking with 5% non-fat milk for 2 h at the room temperature, the membranes were incubated with the following antibodies: MUC1, Occludin, ZO-1, RAC1, p22, KEAP1, NRF2, HMOX1, NQO1, GCLC (Proteintech, USA), and NOX2 (Abcam, UK) overnight at 4 °C. The membranes were washed with TBST thrice for 5 min each time and then incubated with secondary antibodies for 1 h. Finally, the membranes were washed in TBST thrice and subjected to testing by the ECL chemiluminescence detection system (MIniChemi 610, China).

### Quantitative PCR (q-PCR)

RNA from the colonic tissues and cells was extracted using the Trizol Reagent (Tiangen Biotech, China) according to the manufacturer’s protocol. Briefly, 1 mL of the Trizol Reagent was added to the colonic tissues or to the harvested cells and homogenized in a mechanical homogenizer. All polysaccharides were removed by lithium chloride. cDNA was synthesized with the reverse transcriptase kits (Takara, Japan). SYBR Green (Vazyme, China) mix was used to perform the q-PCR analysis, and the data were acquired in the ABI fluorescence quantitative PCR apparatus (7500; USA). The primers used in this experiment are listed in Supplementary Table 1. The respective levels of *β-actin* were used to normalize the target-gene transcription level of each sample.

### Immunofluorescence analysis

The shNC and shQKI5 cells were planted in confocal dishes and then treated with LPS (100 ng/mL) for 24 h. The cells were then washed with PBS, fixed with 4% formaldehyde for 20 min, and permeabilized with 0.5% Triton X-100 for 30 min. The cells were blocked with 5% goat serum for 1 h at 4 °C and incubated with rabbit anti-mouse NRF2 and KEAP1 antibody (Proteintech) for 30 min. The cells were washed and incubated with the DyLight 594 conjugated goat anti-rabbit IgG (Proteintech) for 30 min, and the cell nuclei were counterstained with DAPI (Sigma). The cells were finally observed; their images were captured by microscopy (Olympus IX71) and processed by the cellSens imaging software.

### RNA immunoprecipitation

The control cells (LV-cherry) and QKI5-overexpressed cells (LV-QKI5) were stimulated with LPS (100 ng/mL) for 24 h and then harvested. Using the nuclear protein and cytoplasmic protein extraction kit (Beyotime Biotechnology, China), the nuclear and cytoplasmic proteins were separated. Nuclear proteins were then resuspended in RIPA (Gzscbio, China), to which the rabbit anti-Flag antibody or rabbit IgG (Bethyl Laboratories) was added, followed by co-incubation for 2 h at 4 °C. Next, the protein A/G agarose was added to the supernatants and co-incubated for 2 h at 4 °C. The cells were washed with RIP buffer for five times and incubated with proteinase K (0.2 mg/mL) at 55 °C for 15 min; then, Trizol was added to the cells in order to extract the RNA. Reverse transcription and real-time PCR were then performed with the following specific primers for KEAP1: 5′-CCCGTCAAAGCCCCCCACTGCATAC-3′ (forward) and 5′-TCCAGAGAGGAATGAGTGGCGGATG-3′ (reverse). The RIP results for each of the immunoprecipitation sample were normalized by the level of *β-actin*.

### RNA fluorescence in situ hybridization

The shNC cells and shQKI5 cells were planted in confocal dishes and stimulated by LPS for 24 h, and then hybridized with *Keap1* mRNA interaction probes labeled FITC overnight at 60 °C, followed by incubation with QKI antibody for 12 h. After washing with DEPC water, the cells were stained with fluorescent secondary antibody Alexa 594 and DAPI for 30 min and subjected to microscopy (Olympus IX71) to observe the cells. The *Keap1* mRNA probe sequence was 5′-GAGTTAAGCCGGTTAGTCCCGT-3′.

### Intestinal permeability

FITC-labeled dextran was used to assess the epithelial barrier permeability, as described previously^44^. Briefly, the mice were administered with FITC-dextran (Sigma-Aldrich, 1 g/kg) via oral gavage on day 7 of treatment with DSS. After 6 h, the blood was collected and centrifuged for collecting the plasma, and the plasma FITC-dextran was quantified by fluorescence spectrophotometry.

### 16S rRNA amplicon sequencing and analyses

Each mouse was considered as a separate sample, and each sample weighed at least 1 g. The DNeasyPowerSoil Kit (QIAGEN Inc., Netherlands) was used to extract the total microbial genomic DNA, which was stored at −20 °C before further analysis. The bacterial 16S rRNA genes V4–V5 region was amplified by PCR. After chimera detection, the remaining high-quality sequences were clustered into OTUs at 97% sequence identity by UCLUST (Edgar 2010). A representative sequence was then selected from each OTU based on default parameters. OTU classification approach was performed through BLAST searching of the representative sequences set against the Green genes Database using the best hit.

### Statistics

The data were statistically processed using Prism8 (GraphPad Software, San Diego, CA). All values were expressed as mean ± SEM or SD. All experiments were repeated at least three times independently. Statistical significance between two groups was determined by an unpaired, two-tailed Student’s *t* test. Three or more groups were compared with ANOVA. Multiple comparisons between variables were assessed by one-way ANOVA with Tukey’s multiple comparisons. The microbial data were processed with Permanova test or Mann–Whitney *U* test. *P* < 0.05 was considered to be statistically significant.

## Supplementary information

supplementary figure legends

supplementary figure 1

supplementary figure 2

supplementary figure 3

supplementary figure 4

supplementary figure 5

supplementary table
