# Supplementary material for: Loss of QKI in macrophage aggravates inflammatory bowel disease through amplified ROS signaling and microbiota disproportion
Source: Cell Death Discov. 2021 Mar 23;7:58. doi: 10.1038/s41420-021-00444-w (PMC7988119; doi:10.1038/s41420-021-00444-w)
Supplement: Supplementary file 1 — supplementary figure legends [file 41420_2021_444_MOESM1_ESM.docx]

**Figure S1** Specimens of IBD patients and DSS induced mice model showed QKI was highly co-expressed in the intestinal macrophage cells. Immunofluorescence staining for QKI (green), CD68 (red), and DAPI for nuclei (blue) in colon tissue of active lesion and uninflamed sections from patient with CD (**A**) and UC tissue (**B**). Scale bars: 5000 μm (whole colon section) and 50 μm (enlarged insets). White arrows point to CD68^+^ macrophages with high expression of QKI. (**C**) C57BL/6J mice were divided into two groups and fed with H_2_O and 3% DSS separately. Macrophages of the mice colonic lamina propria were labeled as CD11b^+^F4/80^+^, which gated from CD45^+^ cells by flow cytometry, and QKI expression was detected. Gray: isotype; blue: mice fed with H_2_O; red: mice fed with 3% DSS.

**Figure S2** (**A**) The body weigh change of KO and WT mice fed with H_2_O for 6 days. (n=7/group) (**B**) Gross morphology of colons from WT-H_2_O and KO- H_2_O mice. The length of colon was measured on day 6 (n=7/group). (**C**) Statistical results of colonic length of two groups. (**D**) Representative images of periodic acid-Schiff (PAS)-stained colons and statistical analysis the PAS positive cells number in colon. Scale bars: 200 μm. (**E**) Representative histogram of flow cytometry for CD4^+^ and CD8^+^ T cells in colon tissue of WT-DSS and KO-DSS mice after DSS treatment for 7 days. (**F**) CD4^+^ T cells, Th1 cells (CD4^+^IL-2^+^), Th17 cells (CD4^+^IL-17^+^) and Treg cells (CD4^+^Foxp3^+^) immunostaining. Graph shows qualification of the percentages of Th1, Th17 and Treg cells. All bars represent the mean of measurements from three independent experiments, and the error bars indicate ±SEM. ^*^*P* < 0.05, ^**^*P* < 0.01, ^***^*P* < 0.001, by unpaired, 2-tailed Student’s *t* test (**A, C, F**).

**Figure S3 (A-B)** Representative images of IHC staining of iNOS in colon tissue of CD and UC patients’ specimens. Scale bars: 200 μm and 50 μm (enlarge) (**C**) The percentage of ROS^+^ macrophages collected from CLP of WT-H_2_O and KO- H_2_O groups were detected by flow cytometry and the cells were gated from CD45^+^CD11b^+^. (**D**) Statistical results of macrophages with high level of ROS. (**E**) The ROS level of shNC and shQKI5 cells. Black arrows point the positive cells. Red: ROS probe. Scale bars: 20 μm. (**F**) Statistical results of number of ROS^+^ cells per 100 MODE-K cells. (**G**) MODE-K cells were co-cultured with the supernatant of shNC and shQKI5 cells treated with LPS for 12 h. ROS level of MODE-K cells were detected by ROS probe which show in red. Scale bar, 20 μm. (**H**) Statistical analyses result of ROS^+^ MODE-K cells per 100 cells. All bars represent the mean of measurements from three independent experiments, and the error bars indicate ±SEM. ^*^*P* < 0.05, ^**^*P* < 0.01, ^***^*P* < 0.001, by unpaired, 2-tailed Student’s *t* test (**D, F, H**).

**Figure S4** (**A**) RNA FISH was carried to detect the interaction between QKI protein and *Keap1* mRNA. Control and shQKI5 cells hybridized both with *Keap1* mRNA interaction probes (green) and QKI antibody (red). Nuclei are stained with DAPI (blue). Scale bar: 10 μm. (**B**) Confocal images of shQKI5 cells which stimulated with LPS for 24 h treated or not with BHA (100 μM). NRF2 were shown in red and DAPI in blue. Scale bar: 20 μm. (**C**) ROS level of shQKI5 cells after BHA (500 mM) treatment were detected by ROS probe (red). Scale bar: 50 μm. Black arrows point the positive cells. (**D**) Representative images of IHC staining of iNOS in colon tissue of WT-DSS FMT mice and KO-DSS FMT mice. Scale bars: 50 μm (whole colon sections) and 2­­­­­­0 μm (enlarged insets). (**E**) MODE-K cells were co-cultured with microbiota which derived from KO-DSS mice after BHA treated and then detected the ROS level. (**F**) The image of ROS^+^ MODE-K cells. Scale bar: 50 μm.

**Figure S5** Patterns of microbiota was altered in KO-DSS mice compared to WT-DSS group. (**A**)16S rDNA Shannon diversity index of faeces samples from KO-DSS mice and their control littermates. (**B**) Observed species number in each group. (**C**) Unweighted UniFrac based on clustering tree. (**D**) Bacterial taxonomic profiling in the phylum level of intestinal bacteria from different groups.
