# Supplementary figures and images for: Loss of QKI in macrophage aggravates inflammatory bowel disease through amplified ROS signaling and microbiota disproportion
Source: Cell Death Discov. 2021 Mar 23;7:58. doi: 10.1038/s41420-021-00444-w (PMC7988119; doi:10.1038/s41420-021-00444-w)

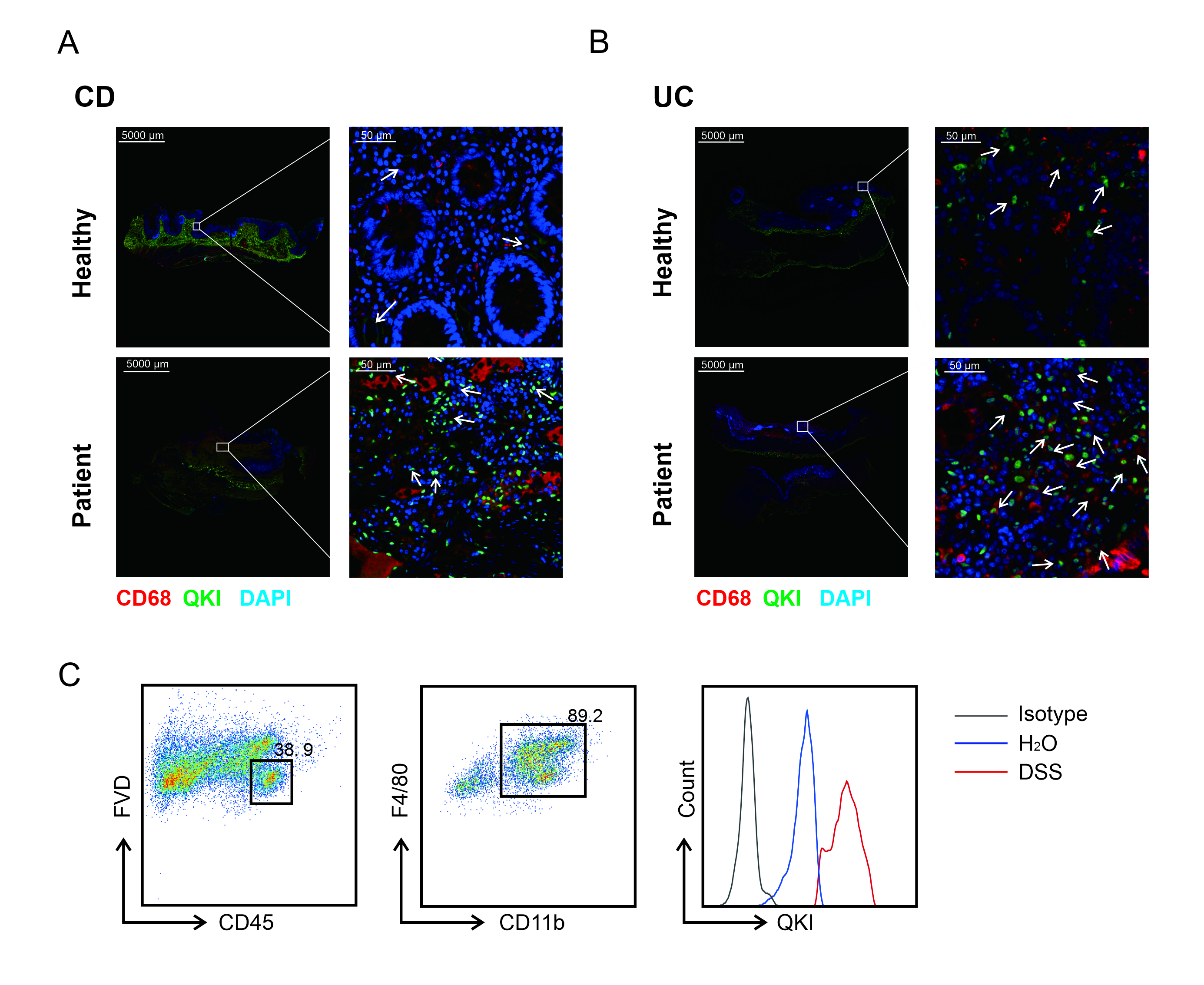

Supplement: Supplementary file 2 — supplementary figure 1 [file 41420_2021_444_MOESM2_ESM.tif]

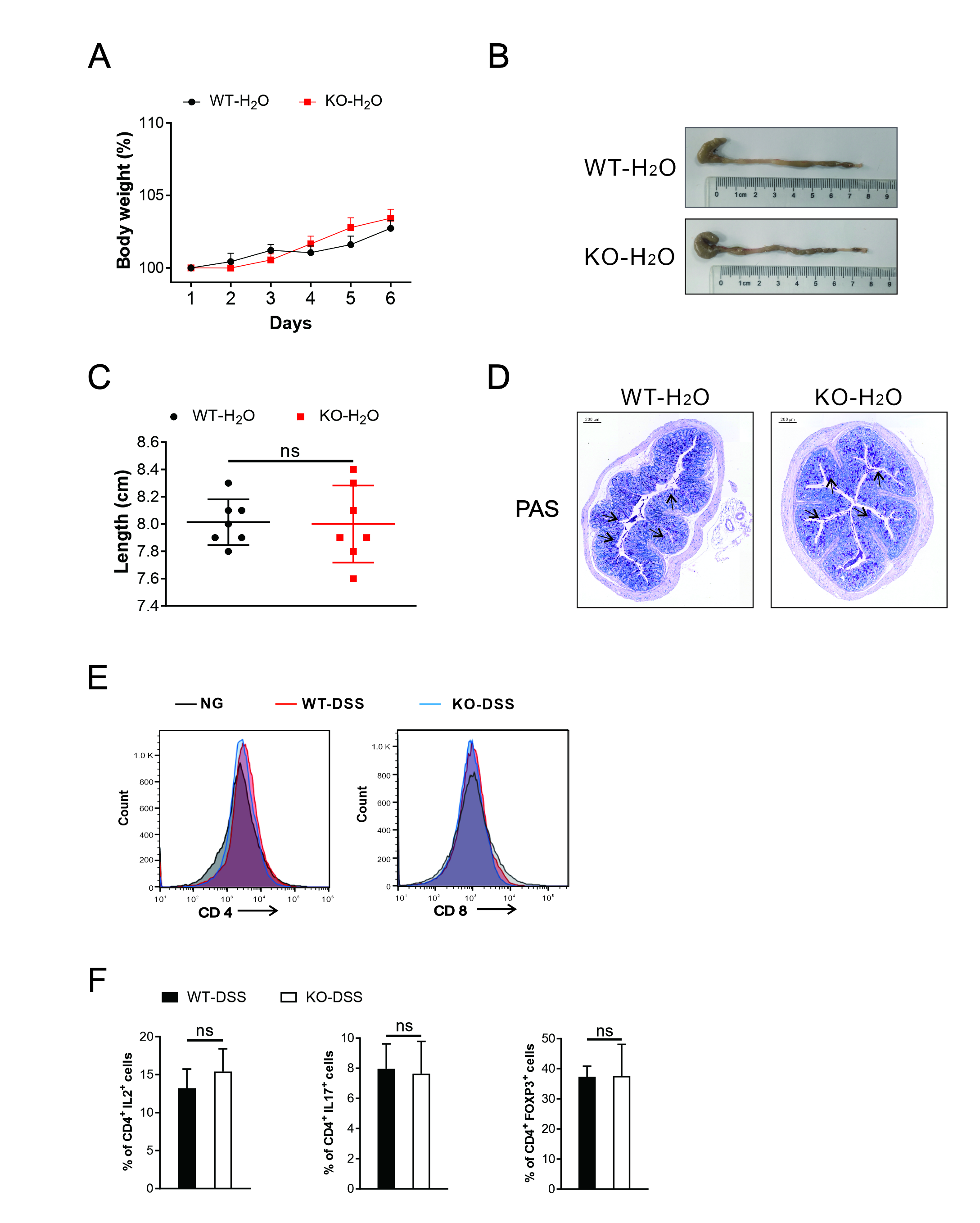

Supplement: Supplementary file 3 — supplementary figure 2 [file 41420_2021_444_MOESM3_ESM.tif]

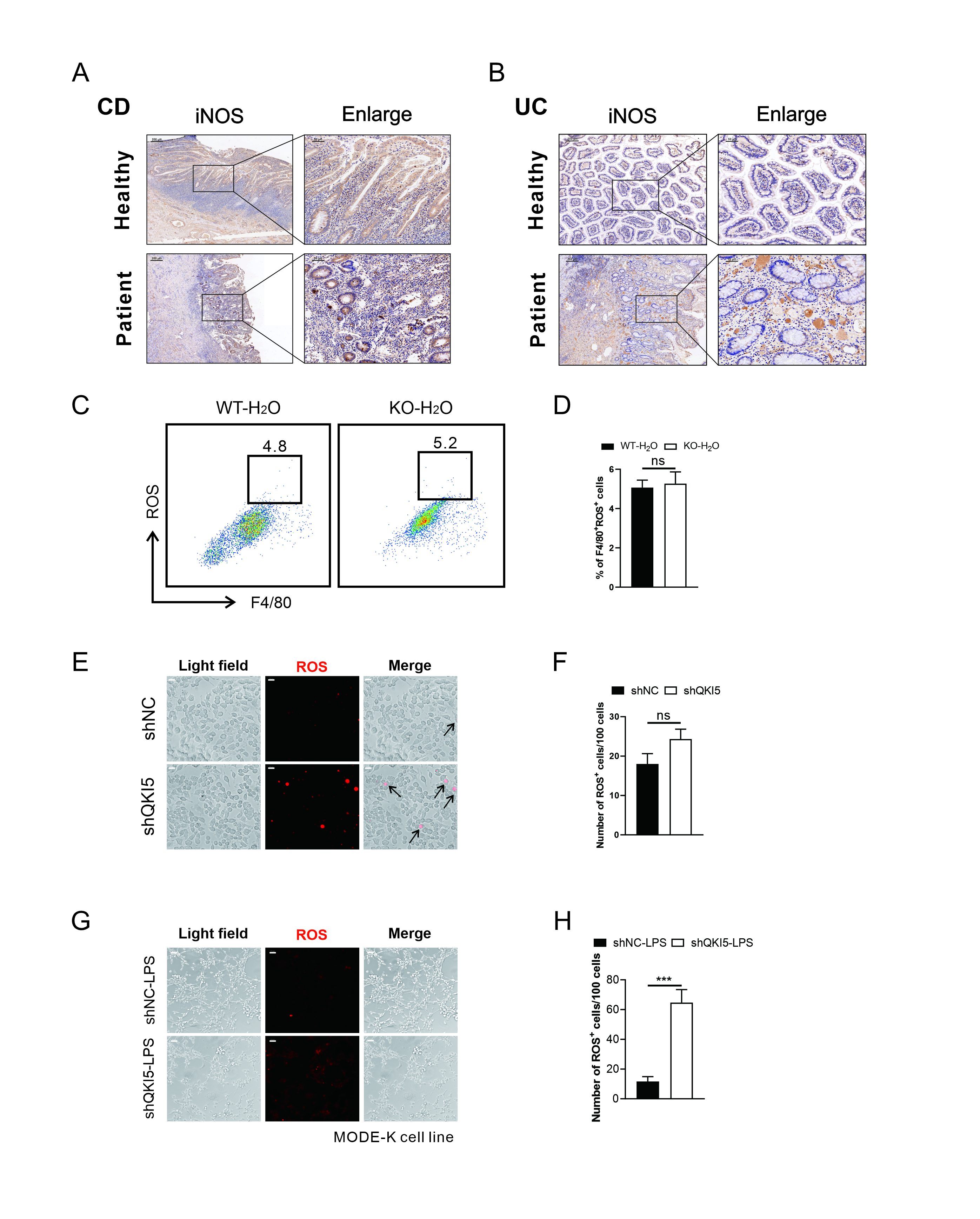

Supplement: Supplementary file 4 — supplementary figure 3 [file 41420_2021_444_MOESM4_ESM.tif]

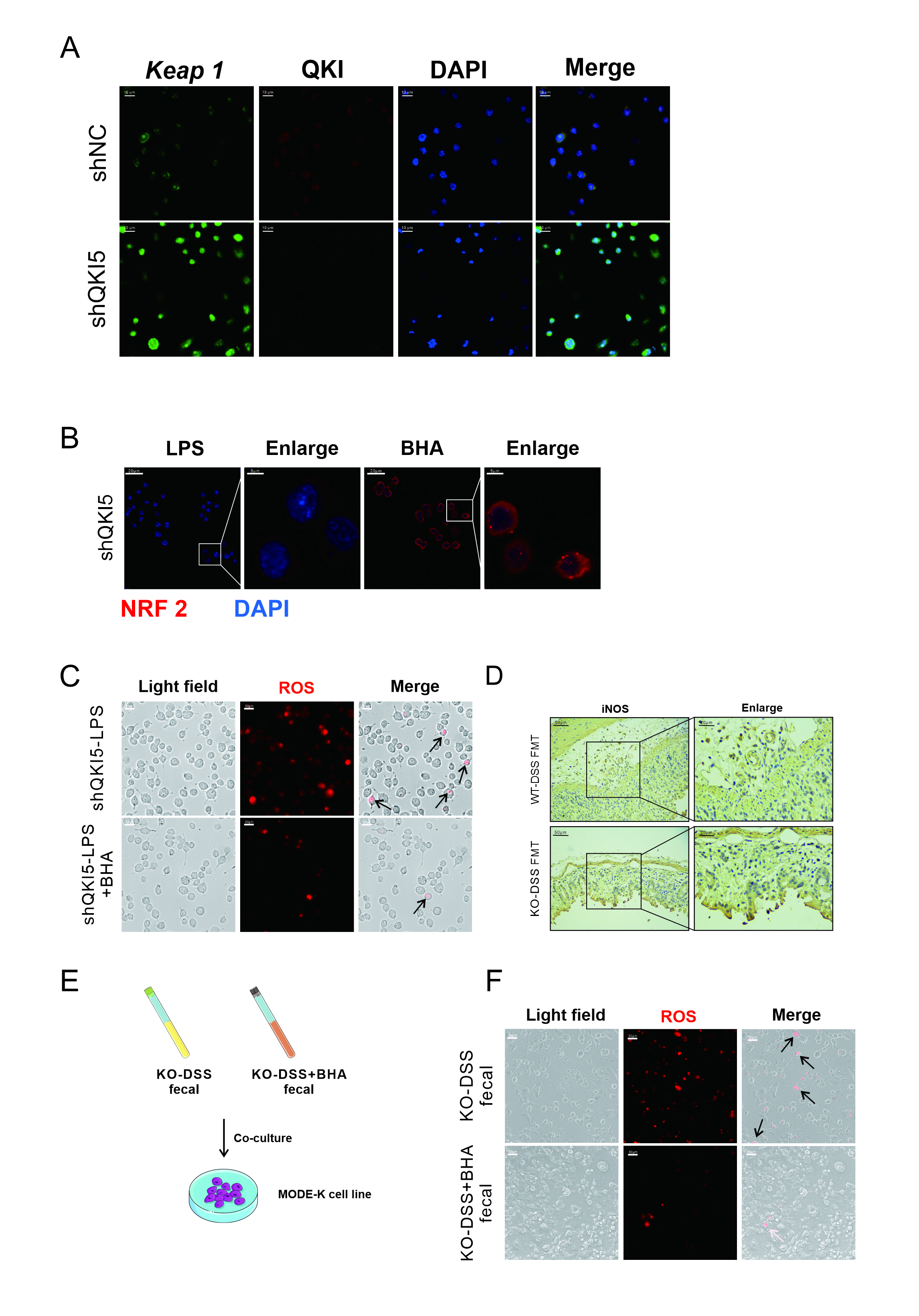

Supplement: Supplementary file 5 — supplementary figure 4 [file 41420_2021_444_MOESM5_ESM.tif]

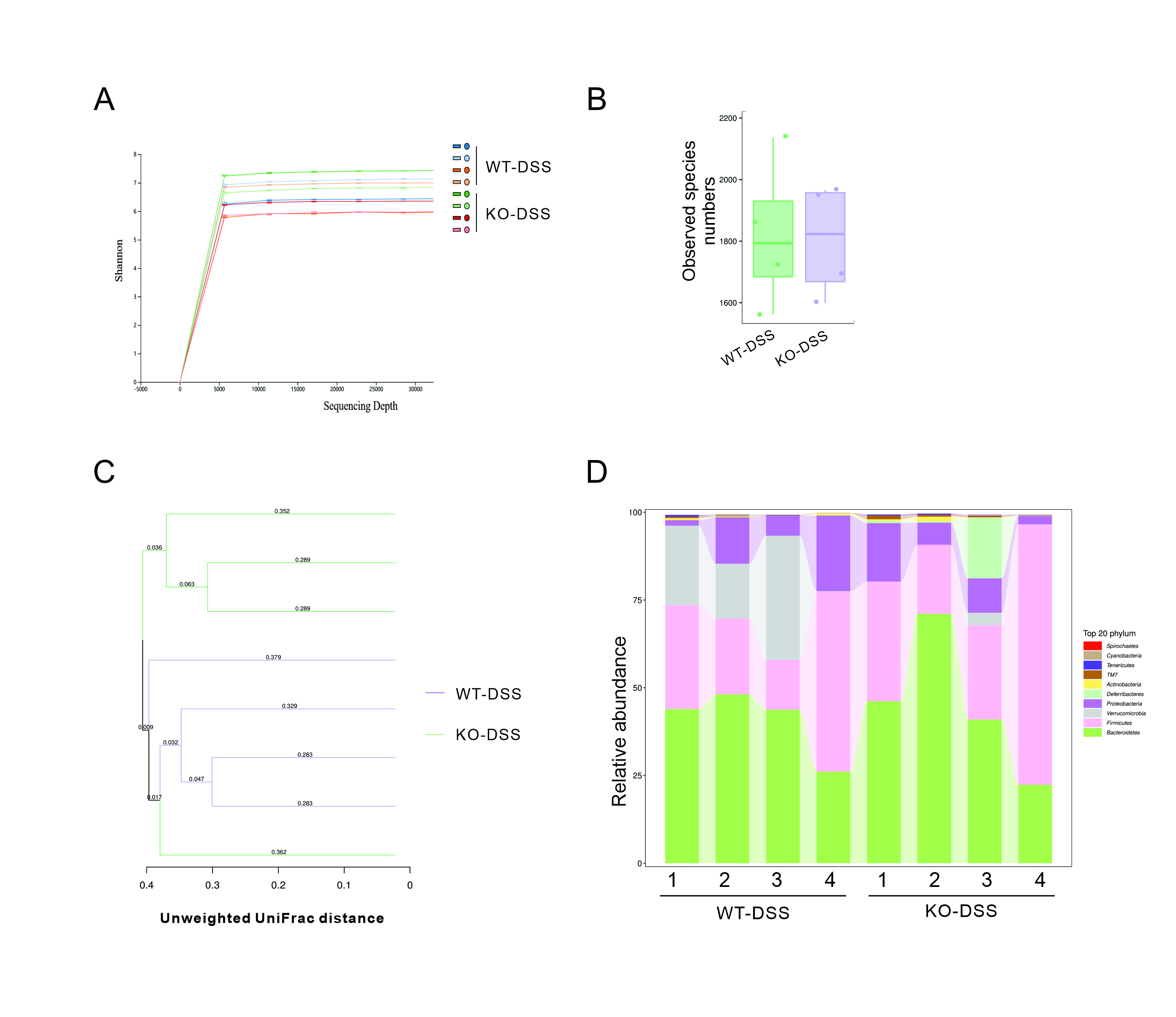

Supplement: Supplementary file 6 — supplementary figure 5 [file 41420_2021_444_MOESM6_ESM.tif]

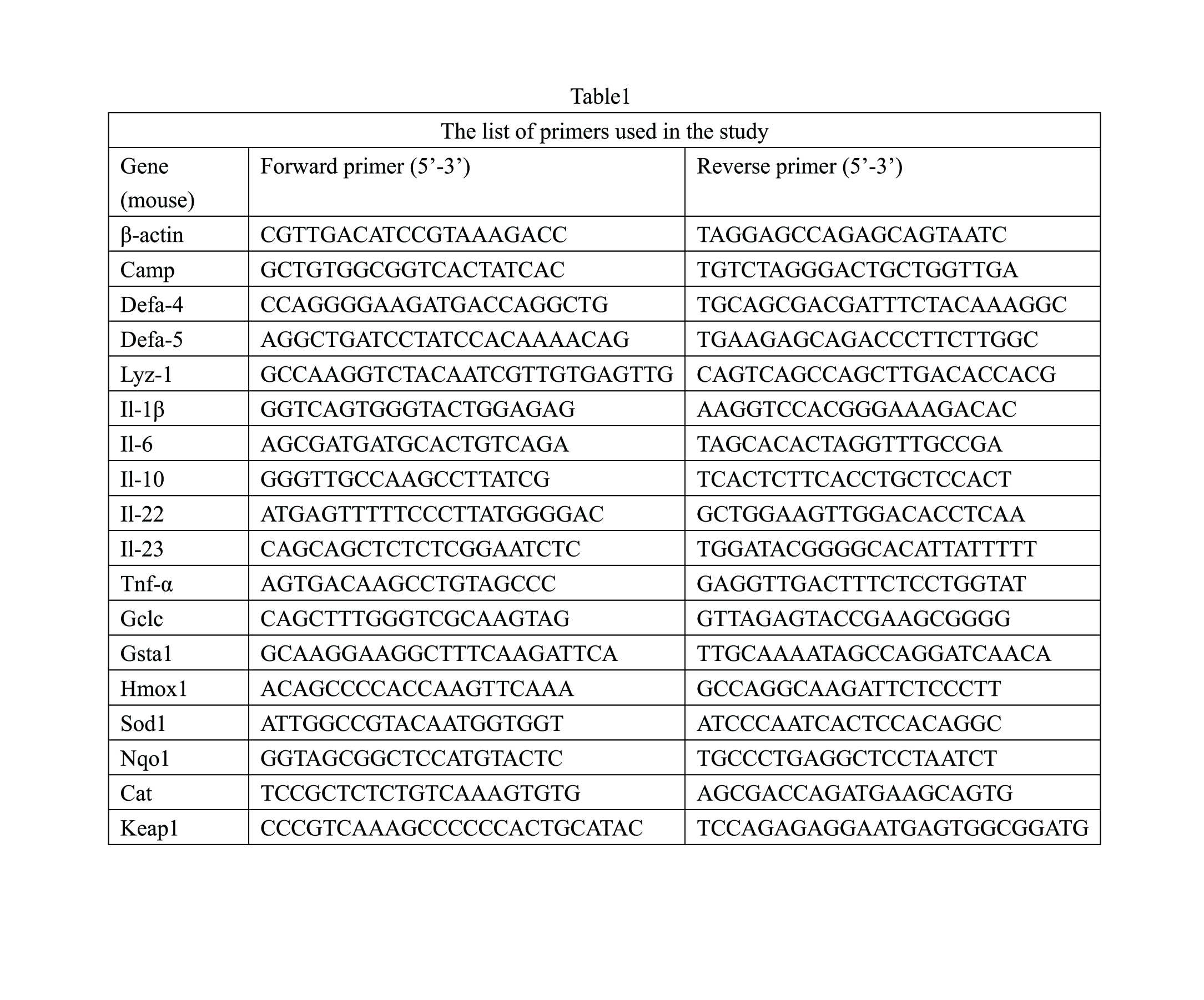

Supplement: Supplementary file 7 — supplementary table [file 41420_2021_444_MOESM7_ESM.tif]
